# Workplace violence in the Psychosocial Care Centers of a city in the state of São Paulo

**DOI:** 10.47626/1679-4435-2021-570

**Published:** 2021-04-30

**Authors:** Aline Bedin-Zanatta, Sérgio Roberto de Lucca, Beatriz Machado-de-Campos Corrêa Silva

**Affiliations:** 1 Departamento de Saúde Coletiva, Universidade Estadual de Campinas, Campinas, SP, Brazil

**Keywords:** health care professionals, Psychosocial Care Centers, workplace violence

## Abstract

**Introduction:**

Workplace violence against health care professionals is a highly prevalent problem and is considered a public health concern by the World Health Organization. Yet most studies on the topic focus on its negative impact on the mental health of workers rather than the causes of these incidents.

**Objectives:**

To describe the frequency of workplace violence and its impact on professionals working in Psychosocial Care Centers (Centros de Atenção Psicossocial [CAPS]) in a large city in the state of São Paulo.

**Methods:**

A quantitative cross-sectional study was conducted on a non-probabilistic sample of 193 health care workers across 11 Psychosocial Care Centers. Participants completed two self-administered instruments: a biosocial questionnaire and the Survey Questionnaire on Workplace Violence.

**Results:**

The results showed that 42.4% of respondents had suffered physical violence; 64.8% had experienced psychological violence; and 29.5% had been victims of bullying/mobbing. In most cases, the victims responded to these incidents by taking no action, asking the perpetrator to stop, or speaking of the incident to a colleague or superior. A verbal warning was issued to perpetrators in only 21% of cases of physical violence. Mobbing had the greatest negative impact on respondents, followed by psychological and physical violence.

**Conclusions:**

Workplace violence is a part of everyday work in Psychosocial Care Centers. Though this violence is often naturalized and considered a collective defense against suffering, it did not prevent participants from reporting high levels of job satisfaction, reflecting the degree to which professionals at Psychosocial Care Centers are committed to their colleagues and to service users, as well as their search for professional recognition.

## INTRODUCTION

Workplace violence can occur in both individual and group settings as the result of the interplay between several different factors; these phenomena have been observed in health care services around the world, and constitute a significant public health concern.^1^ Most cases of workplace violence involve managers who abuse their power by engaging in mobbing. Workers in the service industry, who must deal with customers directly, can also be victims to physical violence at the hands of service users themselves. However, the invisibility of parts of the work process in health care increases the exposure of professionals to many forms of violence that may be hidden in the service relationship.^2^

The buildup of stressors associated with working conditions, the demands of health care work, social issues and the political-economic features of health care systems are among the main triggers of violence in these environments.^1^ Workers in the health care sector are at high risk of exposure to workplace violence due to the intrinsic risks of their activities and their constant contact with service users, with whom they often engage in intense interpersonal interactions.^3^ The extent to which the health of workers is affected by workplace violence, especially in the case of psychological aggression, can be difficult to assess, since victims are often silent in response to these events due to feelings of shame, humiliation or fear. As a result, these incidents can lead to a loss of self-esteem and professional self-confidence, as well as feelings of impotence and dissatisfaction, depression, sexual disorders, absenteeism, and loss of interest in work.^2^

These observations highlight the need for a broader understanding of violence, extending beyond its concrete physical dimension in order to capture its subjectivity and unpredictability.^4^ Individual or collective strategies can be implemented to prevent the suffering associated with workplace violence from turning into an illness.^5^ Public policy on mental health refers to violence as a source of psychological distress associated with barriers to treatment access, institutional mortification, and even the complex issue of substance use.^6^

While a substantial part of the literature on mental health focuses on patients, few studies examine the psychological distress experienced by the health professionals who care for these individuals. The aim of this study was therefore to investigate the perceptions of health care workers regarding the impact of workplace violence in mental health care settings, with a focus on Psychosocial Care Centers (Centros de Atenção Psicossocial; CAPS) in a large city in the state of São Paulo.

## METHOD

### STUDY DESIGN AND LOCATION

An exploratory, cross-sectional quantitative study was conducted in all 11 CAPS in the mental health care network of a city in the state of São Paulo.^7^ The study included all types of CAPS in the region. The 11 centers therefore comprised 6 adult patient services, 3 treatment centers for drug and alcohol abuse, and 2 pediatric centers.

### POPULATION AND SAMPLE: INCLUSION AND EXCLUSION CRITERIA

Participants were selected using non-probabilistic convenience sampling. All individuals at the CAPS during the data collection period were screened for eligibility. The study population consisted of workers of all occupations employed at the CAPS, including physicians, nurses, nursing technicians and assistants, psychologists, managers, occupational therapists, social workers, monitors, pharmacists, pharmacy technicians, physical educators, and speech pathologists.^7^

Inclusion criteria were as follows: having worked at the institution for 6 months or more, working at the CAPS at the time of data collection, providing written consent to participate in the study, and returning a completed questionnaire. Workers who met inclusion criteria were individually approached by the head researcher, informed of the goals of the study, and invited to participate. Participants were then given the research questionnaires in a sealed and coded envelope with instructions on how to complete each instrument. Respondents could choose whether to fill out the questionnaires at their place of work or elsewhere.

Out of an initial pool of 395 CAPS employees, 70 were excluded for working at the institution for less than 6 months; 5 for returning incomplete questionnaires; and 15 for being on medical or maternity leave, leaving a sample of 305 eligible workers. Several of these individuals refused to take part in the study or failed to hand in the questionnaires after at least 5 retrieval attempts, resulting in further sample loss (36.7%; n = 112). The final sample for the study therefore consisted of 193 participants. Fieldwork was conducted from November 2014 to October 2015. Data were collected in the morning, afternoon and night shifts, and continued until all workers had been approached. The head researcher personally delivered and collected the questionnaires individually from each participant.^7^

### STUDY PROTOCOL AND INSTRUMENTS

Two instruments were used for data collection: a biosocial questionnaire developed based on previous studies,^8^ and the *Survey Questionnaire on Workplace Violence in the Health Sector*, developed by the World Health Organization, the International Labor Office, Public Services International and the International Council of Nurses.^3^ After its publication, the instrument was translated and adapted to Portuguese.^9^ The biosocial questionnaire aimed to examine different aspects of the study population. It consisted of a self-report instrument with items investigating the following characteristics: gender, age, marital status, number of children, occupation at the CAPS, time since graduation, education level, length of current employment, history of occupational accidents, medical leave in the past 2 years, job satisfaction and personal relationships.

The *Survey Questionnaire on Workplace Violence in the Health Sector* is also self-administered, and contains 18 questions on physical violence as well as 13 on psychological violence (verbal abuse, bullying/mobbing, sexual harassment and racial discrimination). The instrument also examines the frequency with which the respondent experienced each of type of aggression. The present study focused on incidents involving physical violence, verbal abuse and bullying/mobbing. Physical violence is defined as “the use of physical force against another person or group, that results in physical, sexual or psychological harm. It can include beating, kicking, stabbing, shooting, pushing, biting, and pinching.”^9^ Psychological violence, on the other hand,

is defined as the intentional use of power, including threat of physical force, against another person or group, that can result in harm to physical, mental, spiritual, moral or social development. Psychological violence includes verbal abuse, bullying/mobbing (humiliation/harassment), discrimination and threats.^9^

The assessment of psychological violence is divided in two categories: verbal abuse and bullying/mobbing. The former refers to behavior that humiliates, degrades and disrespects the dignity and worth of an individual. The latter includes offensive and humiliating behavior that disqualifies or demoralizes a person or group, through vindictive, cruel, malicious and repeated attacks.^9^

### STATISTICAL ANALYSIS

The results were analyzed using SPSS, version 2.0. Categorical variables were summarized as ratios and percentages. Significance was set at p < 0.05 with a 95% confidence interval (95%CI). Percentages were compared using Pearson chi-square or Fisher’s exact tests, as appropriate, while differences in means were analyzed using Kruskal-Wallis tests.

A multivariate analysis was performed based on a binary logistic regression model. Logistic regression models including all predictors were constructed, after which variables with p > 0.20 were successively removed using a stepwise approach. Associations between variables were evaluated based on odds ratios (OR) and 95%CI. This project was approved by the Research Ethics Committee of the Faculdade de Ciências Médicas da Universidade Estadual de Campinas (FCM/Unicamp) under protocol number 848.136/2015.

## RESULTS

Most participants in the present study were female (74%) and single (50.5%), with no children (58.3%), a mean age of 35.2 years, a mean of 4.9 years working at the CAPS, and a mean work week of 42.9 hours. The majority of participants worked in nursing (46.9%) and psychology (21%).

Nearly 80% of participants were satisfied with their jobs, reporting high (56.4%) to very high levels (22.2%) of satisfaction. Fifty-eight (30.2%) participants experienced occupational accidents in the year prior to the study, with violence from patients (38 people) identified as the main cause of accidents, followed by sharps injuries (12 people). Interpersonal relationships were described as excellent by 12.4% of participants; good by 60.1% of the sample; fair by 21.7% of participants; and poor or very poor by 5.7% of respondents. The psychological distress experienced by participants manifested indirectly in the form of absenteeism, with 20.7% of respondents having been on medical leave in the previous year, and 57.5% going to work despite being ill, a behavior known as presenteeism.

Most professionals had experienced some form of violence, with psychological violence being the most prevalent (n = 125), followed by physical violence (n = 82) and bullying/mobbing (n = 57). Participants were allowed to provide multiple responses for this item. In most cases, the violence was perpetrated by patients, who accounted for 96.4% of incidents of physical violence, 84.1% of cases of psychological violence and 50.8% of cases of bullying/mobbing. Another interesting finding was that 28% and 35% of participants reported experiencing bullying/mobbing from colleagues and supervisors, respectively.

The most common responses to incidents involving physical violence were asking the person to stop (61%) or trying to defend themselves physically (43.9%). In the case of psychological violence, the most frequent reactions were asking the person to stop (62.4%) and telling a colleague or supervisor (50.4%). When exposed to mobbing, most workers told a colleague (57.9%), asked the perpetrator to stop (43.9%) or reported the incident to a senior staff member (43.9%). Nearly 1/3 of participants had been intimidated or harassed by colleagues or supervisors. In most cases, attackers were issued verbal warnings or faced no consequences.

Table 1 shows the differences in the intensity and frequency of problems resulting from physical violence vs psychological violence; physical violence vs bullying; and psychological violence vs bullying, as well as the psychological impact of these incidents on workers.

**Table 1:** Comparison of the repercussions of different forms of violence, Campinas, state of São Paulo, 2020

Problems experienced	Physical violence vs psychological violence	Physical violence vs bullying/mobbing	Psychological violence vs bullying/mobbing
	p-value*		
Repeated, disturbing memories, thoughts or images of the abuse	0.008^†^	< 0.001^†^	1.15
Avoiding thinking or talking about the abuse, or avoiding having feelings about it	0.09	< 0.001^†^	1.18
Being super-alert, watchful and on guard	0.64	0.021^†^	0.004^†^
Feeling like everything you did was an effort	0.002^†^	< 0.001^†^	0.05^†^

Each problem was rated on a five-point scale, consisting of the following answers: (1) not at all; (2) a little bit; (3) moderately; (4) quite a bit; (5) extremely.

* Student's T test.

† Values considered statistically significant.

Table 2 shows the association between biosocial variables and different types of violence. Statistical tests were used to analyze the association between each variable in the biosocial questionnaire and the experience of different types of violence (physical, psychological, bullying/mobbing). Only variables significantly associated with violence at p < 0.05 were included in the table.

**Table 2:** Association between biosocial variables and different forms of violence in mental health care workers at a CAPS, Campinas, state of São Paulo, 2020

Associated variables	Physical violence	Psychological violence	Bullying/mobbing
Low/very low job satisfaction			0.0168*
Feeling unappreciated			0.0015*
Being currently ill	0.0104*		
Feel underpaid	0.0174*		
Previous occupational accident	< 0.0001*	< 0.0001*	
Working while sick	0.0451*		
Smoking more cigarettes per day		0.0183^†^	
Working as nurse	0.0400^‡^		

* Chi-square test.

† Mann-Whitney U test.

‡ Fisher's exact test.

## DISCUSSION

Physical and psychological violence in the workplace have become global problems with negative consequences for workers and organizations. These incidents have both short- and long-term effects on the relationships between people and their workplace.^1^ Professionals at the CAPS must assume multiple roles and negotiate a wide variety of expectations and subjectivities in the communities where they work.^2,6^ The biosocial profile of professionals in the present study was similar to that observed in a previous investigation of stress, coping and quality of life in health care workers in an outpatient mental health service in the state of São Paulo, where most participants were also female, middle-aged, married, and worked in nursing teams.^10^

The high levels of job satisfaction reported by 80% of participants reflect their commitment to patients and colleagues at the CAPS.^11^ Additionally, many workers reported going to work even when sick, citing their commitment to patients and coworkers as the reason for this behavior (the reason for coming to work while sick was assessed by an open-ended question in the biosocial questionnaire). This pattern, known as presenteeism, reflects the extent to which these workers are engaged with their colleagues and their work. Similar behaviors were observed in a study of a hospital nursing team, where caring for others was viewed as a professional responsibility.^12^

The most frequent forms of violence experienced by participants in the sample were verbal abuse (64.8%), followed by physical violence (42.9%) and bullying/mobbing (29.53%). A study of violence at an emergency service in the city of Porto Alegre^13^ found that 48.7% of workers at the site had experienced verbal abuse, while 24.9% had experienced bullying/mobbing, and 15.2%, physical violence. The main difference between these findings and those of the present study is the frequency of physical violence, which often occurs in situations involving patients with mental illness experiencing acute distress or psychiatric episodes.^14^ It is also possible that, in the CAPS, physical violence is viewed as an expected behavior of acutely ill patients, which tends to be overlooked as it is incorporated and naturalized into the regular work routine.

Unlike physical violence, psychological violence and bullying/mobbing were most often perpetrated by colleagues and supervisors. Passive responses to these incidents, such as not reacting or simply tolerating the abuse, were among the most frequently reported, corroborating the findings of previous studies.^9^ This type of violence had the least consequences for attackers (28%), and was the most likely to have no repercussions at all for perpetrators (71%). This can aggravate feelings of injustice, dissatisfaction and underappreciation at work.

The data in Table 1 reflect the negative impact of psychological violence and the risk of mental illness in health professionals. The psychological violence has long-lasting consequences which tend to be remembered, leading to a negative impact on workers’ psychosomatic resources and contributing to psychological distress, psychosomatic symptoms and mental disorders. These data are in line with the results of a previous study noting that psychological violence can be more harmful to mental health than physical violence.^13^ There was also a significant association between psychological violence and repeated thoughts of the attack; according to previous studies, the persistence and recurrence of these memories contribute to psychological distress and the occurrence of post-traumatic stress (PTSD) and other mental illnesses.^13^^,3)^

### PHYSICAL VIOLENCE

The data shown in Table 2 reveal that the experience of physical violence was associated with feeling underpaid, previous occupational accidents, having an illness at the time of data collection, working while sick in the past year, and working as a nurse.

The perception of a low salary is an objective variable associated with working conditions, and can reflect subjective issues such as feelings of lack of recognition and appreciation for work done, which may be experienced by workers at the CAPS. This perception was also addressed in a study of nurses in a university hospital, which looked into the reasons for their dissatisfaction with their salaries. The authors concluded that participants did not feel the salary met their personal and professional needs, and was incompatible with their attributions, responsibilities and workload.^15^ This may explain the association between a history of physical violence and dissatisfaction with salary from a symbolic rather than a strictly financial standpoint: professionals who have experienced violence may feel that this was not part of their job description and that the complexity of their role is not being recognized. This is likely to be the case in settings such as the CAPS, where workers spend long periods in contact with the intersubjective world of patients with mental illness, accompanying each patient over the course of their lives. As a result, highly committed professionals expect to be recognized for their work. Job satisfaction is a work-related variable related to objective (salary) and subjective (recognition) compensation. Therefore, recognition by colleagues and superiors may be a protective factor against mental illness in health workers.^5^

The experience of physical and psychological violence was also associated with a history of occupational accidents. Health professionals must receive information and training on the different types and sources of workplace violence, and every form of violence in the workplace must be reported. Health care workers must encourage colleagues who suffered acts of violence to report these incidents as soon as possible. All reports should protect the confidentiality of the professionals and allow for an analysis of the root cause of each incident in order to contribute to prevention efforts.^1^ Therefore, cases of violence should be investigated in a way that allows for the determination of the causes of each incident, taking care not to blame or judge the victim. Researchers have found that the conditions of the production process must be investigated, and occupational accidents must be viewed as health issues arising from the social relationships established in these settings^16^ rather than the result of attitudes and behaviors of individual workers. Reporting is a crucial part of resolving this issue, since there is a period of latency between the traumatic event and the onset of trauma-related symptoms, especially for mental illnesses and PTSD, which manifest 3 or more months after the date of the incident. Studies show that 40 to 70% of people who experience a traumatic event develop symptoms of PTSD.^1,17^

Having an illness at the time of the study and working while ill in the previous year were associated with perceived health status, suggesting that victims of violence may be more vulnerable to illness.^17^ The extent to which physical violence affects nurses has been extensively examined, with several studies noting that these individuals are among the most vulnerable to violence among all health care occupations.^1,13,18^ This may be because nursing professionals are directly involved in patient care for all 24 hours of the day, and as such, are exposed to high-risk situations and susceptible to violent behavior by acutely ill patients, in addition to spending more time with patients, accompanying them in simple to complex interventions.

The logistic regression model predicting a history of physical violence based on biosocial variables revealed that, after the necessary adjustments, CAPS workers who experienced physical violence were 14.32 times more likely (OR = 14.32) to have average to low levels of job satisfaction.

### PSYCHOLOGICAL VIOLENCE

Experiencing psychological violence was associated with a higher likelihood of having a work accident in the previous year and smoking a higher number of cigarettes. Studies showing the repercussions of psychological violence on workers’ physical and mental health find that these may include antisocial behaviors such as smoking or engaging in alcohol abuse.^11,19^ The habit of smoking in the CAPS is part of a complex set of interactions. A qualitative study performed in a CAPS in Rio de Janeiro showed that workers used smoking as an individual defense against suffering. Attitudes such as going out to smoke or eat during team/supervisor meetings were most common strategies or resources used by professionals to avoid stressful situations at work.^20^

Occupational accidents may also be a consequence of psychological violence. This type of abuse can contribute to poor concentration which, in turn, could lead to occupational accidents. The final predictive model for psychological violence revealed that CAPS workers with such a history were 3.2 times more likely (OR = 3.2) to have suffered an occupational accident.

### BULLYING/MOBBING

Bullying/mobbing were significantly associated with low to very low job satisfaction and feelings of underappreciation. This reinforces the fact that the repercussions of workplace violence extend far beyond the attacks themselves, ultimately affecting the extent to which workers feel appreciated.^13^ Participants who suffered bullying/mobbing had lower levels of job satisfaction and felt less valued. Both horizontal and vertical mobbing, perpetrated by colleagues and superiors, respectively, have devastating effects on the victims, as such incidents often take the form of structural violence. The lack of an institutional response to these situations constitutes an ethical problem, as it leads victims to feel silenced and blamed. This perverse dynamic may result in feelings of isolation and distress, leading victims to become ill or quit their jobs.^21^ The final predictive model of mobbing and biosocial variables revealed that workers at the CAPS who suffered mobbing were 3.39 times more likely (OR = 3.39) to have regular or low levels of job satisfaction.

In the modern world, the process whereby work generates different forms and manifestations of violence results in subjectivation and precarization. Acts of intolerance experienced at work can be assimilated into workers’ subjective representation and memory, giving us a window into their experiences through the traces, gestures, subtleties and memories preserved by workers over time.^22^ Since few studies have been performed in other CAPS, the present findings were compared to those of investigations conducted in other professional categories. The breadth of this study, which included all 11 CAPS in a large town, allowed for a reliable assessment of the object of study, capturing the complexity and different conceptions of the phenomenon of violence.

The dynamics of the setting and the occupational overload of professionals at the CAPS may have interfered with their ability to complete the questionnaires during work hours and at the service itself, which may have compromised the sample. Limitations of this study include the heterogeneity of the sample, as reflected by the variability in occupational representation, as observed in the case of psychologists, and the cross-sectional design, which prevented the identification of causal relationships between variables. Furthermore, the results of the present study may not be generalized to CAPS in other cities.

In conclusion, professionals working at the CAPS in the city studied were mostly female, young, single, with no children, and satisfied with their jobs and interpersonal relationships. Nevertheless, nearly half the sample had experienced some form of physical violence. Most had suffered verbal abuse and nearly 1/3 had been victims of mobbing, with most attacks reportedly perpetrated by patients. The following associations were observed between variables: physical violence was associated with being currently ill, perceiving their pay as low, previous occupational accidents, having worked while sick in the previous year and being a nurse; psychological aggression was associated with previous occupational accidents and number of cigarettes smoked a day; and bullying/mobbing were associated with low to very low job satisfaction and feelings of underappreciation.

The work of professionals at the CAPS revolves entirely around the provision of care, and inevitably broaches issues associated with sexual, intrafamiliar, and other forms of violence, in addition to the neglect of service users by public organizations and society as a whole. The constant contact with the reality of each patient can generate feelings of impotence and ultimately affect workers’ professional realization. The experience of physical violence is often naturalized by CAPS workers. This behavior can be interpreted as a collective defense strategy against the suffering experienced in their everyday work. Victims of bullying/mobbing showed signs of emotional scarring, with negative consequences on their work and subjectivity. The provision of care at the CAPS occurs in a dynamic setting, permeated by individual, social, historical, territorial and symbolic relationships. Under these circumstances, it is challenging to be a caretaker. Despite the different forms of violence experienced by workers, they feel underappreciated, which contributes to their psychological suffering and their own need of assistance.
